# Cross-cultural adaptation and psychometric properties of the Brazilian-Portuguese version of the Quality of Prenatal Care Questionnaire (QPCQ)

**DOI:** 10.11606/S1518-8787.2019053000565

**Published:** 2018-12-20

**Authors:** Rodrigo Dias Nunes, Gabriel Cremona Parma, Andressa Cardoso de Campos, Paula Locatelli, Jefferson Traebert

**Affiliations:** I Universidade do Sul de Santa Catarina. Faculdade de Medicina. Palhoça, SC, Brasil; II Universidade do Sul de Santa Catarina. Programa de Pós-Graduação em Ciências da Saúde. Palhoça, SC, Brasil

**Keywords:** Prenatal Care, psychology, Psychometrics, Surveys and Questionnaires, utilization, Translations, Health Care Quality, Access, and Evaluation

## Abstract

**OBJECTIVE:**

To translate and to observe the psychometric measures of the Brazilian version of the Quality of Prenatal Care Questionnaire.

**METHODS:**

The translation protocol followed the standards of the International Society for Pharmacoeconomics and Outcomes Research. Descriptive statistics were performed to identify characteristics of 280 literate postpartum women in a public hospital. We examined the internal consistency using Cronbach's alfa. To determine the test-retest reproducibility and the instrument's stability, we performed the intraclass correlation coefficient and Bland and Altman plot between two applications. We investigated the item's properties using the item response theory.

**RESULTS:**

The overall Cronbach's alpha index was 0.975. The intraclass correlation coefficient was 0.995 (95%CI 0.993-0.996) and a uniform distribution was visualized at the Bland and Altman plot. The item response theory identified the discriminatory power and the difficulty level of the instrument and of each item. The instrument showed acute angulation of the expected total score, and good concentrate information and good standard error curves, preserving the latent construct and its original items.

**CONCLUSIONS:**

This analysis concluded that the Brazilian version of the Quality of Prenatal Care Questionnaire is a high-quality, reliable and valid questionnaire to determine the quality of prenatal care among Brazilian women. The questionnaire is suitable for the cultural context represented.

## INTRODUCTION

The World Health Organization (WHO) has been aiming, since 2015, for a world in which all pregnant women and their newborns can receive quality care for the whole pregnancy, childbirth and puerperium, as one of the goals for the millennium^1^ ^,^ ^2^. In this context, the low quality of prenatal care in some services becomes an obstacle to the reduction of the rates of preventable mortality and morbidity^3^ ^-^ ^6^. The quality of the prenatal care in health services is based on several characteristics such as physical infrastructure, management and human resources, ability and capacity of the health providers to care for a woman during her gestation period, as well as the social and cultural difficulties inherent in each country or region^7^ ^-^ ^10^.

Some authors consider as prenatal care of quality, the follow-up at the early beginning of the pregnancy, which is until the 12th week of gestation, and the number of prenatal consultations performed^11^ ^,^ ^12^. To achieve the objectives of quality prenatal care, the Brazilian Ministry of Health (MS) has determined that ensuring a minimum of six consultations, carrying out educational activities and making available all the basic and complementary exams considered essential for the gestational period is crucial. It also expects to correctly maintain the pregnant woman's card, which includes all records of consultations and conducts^13^ ^,^ ^14^. The prenatal care must allow the linkage of an adequate outpatient service and hospital care, guaranteeing cozy consultations, without unnecessary interventions, using appropriate instruments and with trained professional staff^13^.

However, recent studies show aiming the quality of prenatal care, the attention and guidance provided to the patients, and the quantity and quality of time available to them can be more important than the quantification of caregiving^15^ ^-^ ^18^. Researchers from McMaster University, Canada, conducted a study to evaluate the quality of prenatal care, using subjective parameters related to patient's perception, to stimulate the involvement of the pregnant woman in her own prenatal assistance^19^. Interviewing pregnant women and their attending professionals, clinical care and interpersonal relationships stood out as essential to the quality of care, surpassing the care structure.

The Quality of Prenatal Care Questionnaire (QPCQ)^20^ was developed as result of a rigorous process of psychometric creation and testing. The QPCQ is a self-responsive instrument that measures the overall quality of prenatal care, divided into the six following factors: Information Sharing, Anticipatory Guidance, Sufficient Time, Accessibility, Availability, and Support and Respect. The QPCQ has been translated into several languages, but only validated in a few cultural contexts as Canada^20^, Australia^21^, France^22^, Myanmar^23^ and Portugal^23^.

This instrument, validated and reliable, can be easily used in the scientific environment, especially in studies that seek to evaluate the perceptions of women about the quality of prenatal care. It can also be used to compare the quality of care in different regions, populations, types of health care provider and service delivery models. Epidemiological studies can also evaluate the relationship between prenatal care quality and maternal and infant outcomes^19^ ^-^ ^23^.

This QPCQ in Portuguese (Brazil) can be an evaluation resource in health systems, to improve them, following the path of the development of WHO goals for millennium. This study aimed to perform cross-cultural adaptation and psychometric properties of the QPCQ assessment instrument for the Portuguese language in the Brazilian cultural context, providing a research instrument in Portuguese language and contributing to a scientific analysis related to prenatal care.

## METHODS

This study consisted of cross-cultural adaptation and psychometric testing. The original version of the QCPQ was developed by Sword et al. at the School of Nursing of the McMaster University, Canada^19^ ^,^ ^20^. As QCPQ contains 46 questions, we considered a minimum proportion of five patients for each question^24^. For the minimum sample size of 230 patients, 20% was added to account for possible losses. Based on that criterion, the minimum sample size required was of 253 puerperal patients.

The data were collected from the public Maternity of the Regional Hospital in the city of São José, state of Santa Catarina, Brazil, from February to May 2017. Patients hospitalized at the puerperium ward who had singletons, eighteen or more years old, at least three prenatal appointments and ability to read and write in Portuguese were included. Patients with any psychiatric disorders that could preclude their participation in responding the questionnaire, and those who conceived stillbirth or newborn with neonatal death during the admission period were excluded.

All patients were invited and data were collected by interviews with the pregnant women, performed by two third-year resident physicians of gynecology and obstetrics, during the daily routine with those women who consented to participate. The interview instrument contained the questions of the Brazilian version of CPCQ in addition to sociodemographic issues: age in full years; self-reported skin color; whether or not she was living with a partner at that time; years of study completed. In addition, previous obstetric questions such as parity, number of previous vaginal births, cesareans and abortions were also asked. Information regarding delivery route and the newborn weight was collected from the medical chart. The guidelines followed in this study were performed according to those proposed by Beaton et al and Wild et al.^25^, according to the International Society for Pharmacoeconomics and Outcomes Research (ISPOR), respecting the following stages: Stage I: Initial translation into Brazilian Portuguese. Stage II: Synthesis of the translations. Stage III: Back translation. Stage IV: Committee of specialists. Stage V: Test of the prefinal version. Stage VI: Submission of the results of the pretest to the researchers.

Descriptive statistics were performed to identify the sociodemographic (age, skin color, living with partner and schooling) and obstetric (gestational age, delivery route, parity and newborn weight) characteristics of the study participants and to determine subscale means and standard deviations. The means and standard deviations of the factors obtained from the two applications were calculated.

The internal consistency was examined using general Cronbach-alfa. Its reliability was also calculated with the correlation of each question with the overall result of the Brazilian version of the QPCQ and by factors of the original questionnaire. In addition, the value of the Cronbach-alfa was calculated for each item in case any would have to be excluded from the Brazilian version of the QPCQ. We performed the previous analyses using the software SPSS 18.0.

The test-retest reliability, which is the consistency of measurements made with the same instrument, at different times, was performed with all the 280 patients who participate in the study, both with the same criteria and by the same physician. The interval between the two interviews ranged from three to four days, within the hospitalization period. The intraclass correlation coefficient and a Bland and Altman plot between both applications were performed to determine the test-retest reproducibility and the instrument's stability.

To observe the adequacy of the exploratory factor analysis against the data, the correlation matrix between each pair of questions was analyzed using Pearson's linear correlation. The overall adequacy of the exploratory factorial analysis was also assessed using the Kaiser-Meyer-Olkin (KMO) and Bartlett sphericity tests.

Commonalities analysis was performed to define the quantity of factors that could represent the structure of the original variables. To define the number of factors, we used the Kaiser-Guttman criterion of latent dimensions, only with the factors corresponding to eigenvalues greater than one or very close to one (λ > 1). To facilitate the interpretation of the factors, the extraction method of main components was accomplished using the factor rotation by the Varimax method. We performed the previous analyses using the software SPSS 18.0.

To define the number of factors that could represent the structure of the original variables, a Scree plot test was performed. The unidimensionality of the instrument allowed investigating the item's properties, individually, and the overall construct validity, by the item response theory (IRT). It was performed with the software R 3.30, within the package named “graded”, which was selected after the ANOVA analysis. The Patient-Reported Outcomes Measurement Information System (PROMIS)^25^ group recommends the IRT to test the item-level. We used the IRT to evaluate the 46 items, to estimate the latent trait value, and to create the measurement scale. The analysis of the IRT identified the discriminatory power of the instrument and of each item.

The Ethics Committee on Human Research of the Universidade do Sul de Santa Catarina, Brazil, approved the project under CAA 58099616.0.0000.5369. All the subjects voluntarily signed an informed consent form, written according to the standards of the Declaration of Helsinki. The authors of the original instrument authorized the entire process, from translation to transcultural adaptation of the QPCQ into the Portuguese language in the context of the Brazilian culture.

## RESULTS

To compose the study, 295 women were recruited. In 10 cases, collecting the second questionnaire was not possible, because they were discharged before the interval required for the retest, and five patients decided to abandon the study before responding to the first questionnaire, which was considered refusal, totaling 280 women participants.

Their age ranged from 18 to 39 years, with an average of 26.2 (± 5.8) years old. Gestational age at birth ranged from 34 to 41 complete weeks of gestation, with a mean of 39 ± 1 weeks. All the women were Brazilian, spoke Portuguese as a native language, and performed the prenatal follow-up in the public health network by doctors or nurses. Table 1 describes other sociodemographic and obstetric characteristics.

**Table 1:** Characteristics of the study participants. Hospital Regional de São José, Santa Catarina, Brazil, 2017. (n = 280)

Characteristics		Distribution n (%)	95%CI
Skin color			
	White	228 (81.4)	76.8-86.0
	Non-white	52 (18.6)	14.0-23.2
Living with partner			
	Yes	232 (82.9)	78.5-87.3
	No	48 (17.1)	12.7-21.5
Schooling (years completed)			
	Up to 8	162 (57.9)	52.1-63.7
	> 8	118 (42.1)	36.3-47.9
Delivery route			
	Vaginal	220 (78.6)	73.8-83.4
	Cesarean	60 (21.4)	16.6-26.2
Parity			
	Primipara	120 (42.9)	37.1-48.7
	Multipara	160 (57.1)	51.3-62.9
Previous vaginal births			
	0	149 (53.2)	47.4-59.0
	1	74 (26.4)	21.2-31.6
	2	30 (10.7)	7.1-14.3
	≥ 3	27 (9.6)	6.2-13.0
Previous cesareans			
	0	227 (81.1)	76.5-85.7
	1	42 (15.0)	10.8-19.2
	2	7 (2.5)	0.7-4.3
	≥ 3	4 (1.4)	0.0-2.8
Previous abortions			
	0	227 (81.1)	76.5-85.7
	1	41 (14.6)	10.5-18.7
	2	10 (3.6)	1.4-5.8
	≥ 3	2 (0.7)	0.0-1.7
Newborn weight (grams)			
	< 2,500	16 (5.7)	3.0-8.4
	2,500-4,000	252 (90.0)	86.5-93.5
	> 4,000	12 (4.3)	1.9-6.7

The mean scores of the factors obtained from both applications ranged from 2.20 to 3.42 and 2.26 to 3.39, respectively, out of a total score of five. The factor Availability had the lowest mean rating, while Approachability had the highest mean rating at both moments when the QPCQ was applied.

The reliability analysis of the first application of the QPCQ presented an overall Cronbach's alpha index of 0.975. The Cronbach-s alpha index of each item was deleted from subscale and remained similar to the overall index. Each factor evidenced the same internal consistency reliability. Table 2 shows the distribution of the corrected item-total subscale correlation and the Cronbach's alpha for each subscale.

**Table 2:** Reliability analysis by the Item Response Theory of the Brazilian version of the QPCQ. Hospital Regional de São José, Santa Catarina, Brazil, 2017. (n = 280)

Factor (Subscale) items of Brazilian version of the QPCQ		Cronbach's alpha		Item response theory	
				a*	b**
Factor 1 - Information Sharing (9 items) Cronbach's alpha = 0.920					
3	I was given adequate information about prenatal tests and procedures	0.743		2.805	-0.292
6	I was always given honest answers to my questions	0.628		1.800	-0.691
11	Everyone involved in my prenatal care received the important information about me	0.796		3.179	0.717
17	I was screened adequately for potential problems with my pregnancy	0.762		3.006	0.022
22	The results of tests were explained to me in a way I could understand	0.723		2.509	1.447
33	My prenatal care provider(s) gave straightforward answers to my questions	0.798		3.603	-0.412
39	My prenatal care provider(s) gave me enough information to make decisions for myself	0.703		2.540	1.534
43	My prenatal care provider(s) kept my information confidential	0.549		1.453	-0.179
45	I fully understood the reasons for blood work and other tests my prenatal care provider(s) ordered for me	0.703		2.449	-0.289
Factor 2 - Anticipatory Guidance (11 items) Cronbach's alpha = 0.876					
2	My prenatal care provider(s) gave me options for my birth experience	0.832		4.109	-0.612
4	I was given enough information to meet my needs about breastfeeding	0.475		1.307	0.606
10	My prenatal care provider(s) prepared me for my birth experience	0.806		4.627	0.393
13	My prenatal care provider(s) spent time talking with me about my expectations for labor and delivery	0.774		2.783	-1.086
16	I was given enough information about the safety of moderate exercise during pregnancy	0.723		2.176	-0.628
20	I received adequate information about my diet during pregnancy	0.445		1.108	0.863
24	My prenatal care provider(s) was interested in how my pregnancy was affecting my life	0.740		3.119	0.596
27	I was linked to programs in the community that were helpful to me	0.375		0.737	-1.879
31	I received adequate information about alcohol use during pregnancy	0.401		0.944	1.581
42	I was given adequate information about depression in pregnancy	0.468		1.065	-2.408
46	My prenatal care provider(s) took time to ask about things that were important to me	0.726		2.585	-1.180
Factor 3 - Sufficient Time (5 items) Cronbach's alpha = 0.863					
1	I had as much time with my prenatal care provider(s) as I needed	0.678		1.922	-0.827
8	My prenatal care provider(s) was rushed	0.619		1.627	0.408
18	My prenatal care provider(s) always had time to answer my questions	0.808		3.126	-2.378
30	My prenatal care provider(s) made time for me to talk	0.747		2.298	-0.661
44	My prenatal care provider(s) took time to listen	0.714		2.228	-0.465
Factor 4 - Approachability (4 items) Cronbach's alpha = 0.778					
15	My prenatal care provider(s) was abrupt with me	0.444		1.158	2.153
23	I was rushed during my prenatal care visits	0.692		2.201	-0.089
28	My prenatal care provider(s) made me feel like I was wasting their time	0.583		1.383	-0.103
40	I was afraid to ask my prenatal care provider(s) questions	0.645		1.678	2.006
Factor 5 - Availability (5 items) Cronbach's alpha = 0.843					
9	I knew how to get in touch with my prenatal care provider(s)	0.613		1.413	-0.728
12	Someone in my prenatal care provider(s)'s office always returned my calls	0.587		1.374	-2.666
32	My prenatal care provider(s) was available when I had questions or concerns	0.692		1.935	0.175
35	I could always reach someone in the office/clinic if I needed something	0.576		1.272	-0.476
38	I could reach my prenatal care provider(s) by phone when necessary	0.618		1.540	-1.646
Factor 6 - Support and Respect (12 items) Cronbach's alpha = 0.938					
5	My prenatal care provider(s) respected me	0.402		1.063	0.892
7	My prenatal care provider(s) respected my knowledge and experience	0.746		3.097	0.534
14	My decisions were respected by my prenatal care provider(s)	0.843		4.786	1.915
19	My prenatal care provider(s) was patient	0.601		1.575	-0.198
21	I was supported by my prenatal care provider(s) in doing what I felt was right for me	0.825		4.742	-2.214
25	My prenatal care provider(s) supported me	0.753		3.191	-0.567
26	My prenatal care provider(s) paid close attention when I was speaking	0.750		2.316	0.203
29	My concerns were taken seriously	0.736		2.647	1.018
34	I was in control of the decisions being made about my prenatal care	0.788		3.630	-1.887
36	My prenatal care provider(s) supported my decisions	0.808		3.938	-0.106
37	I was at ease with my prenatal care provider(s)	0.716		2.485	1.042
41	My values and beliefs were respected by my prenatal care provider(s)	0.727		2.804	-0.476

* Parameter a: discrimination of the item.

** Parameter b: mean difficulty of the item.

The intraclass correlation coefficient between the two questionnaires presented R = 0.995 (95%CI 0.993-0.996). The correlation of the differences between both applications and the means of the final results were well distributed at the Bland and Altman plot.

The analysis of the correlation matrix showed a linear correlation between most of the questions (p < 0.001), in which Pearson correlation coefficients were higher than 0.3. These measures proved the adequacy of the exploratory factorial analysis of the Brazilian version of the instrument evaluated. The KMO measure of sampling adequacy was 0.934, evidencing a correlation between the variables. The Bartlett sphericity test also showed adequacy of the factorial analysis technique (p < 0.001).

**Figure 1:**
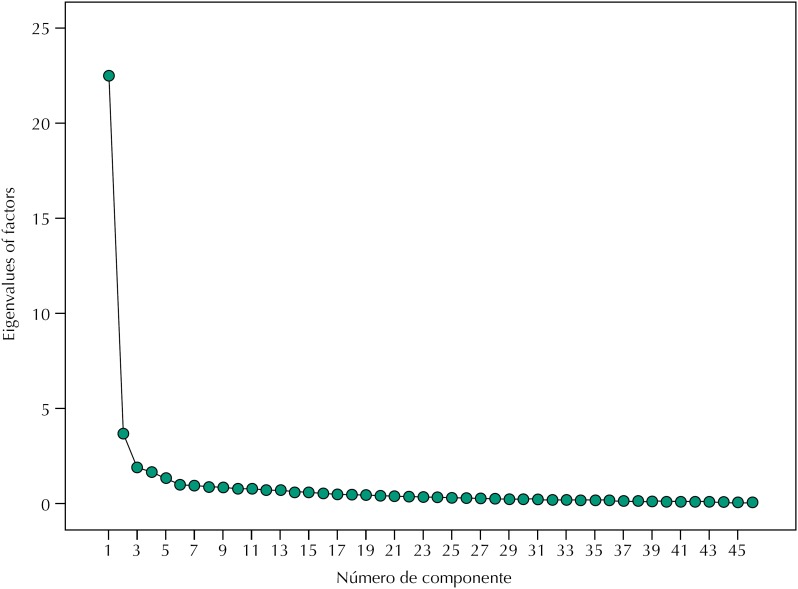
Scree plot of latent factors values according to extraction order of the Brazilian version of the QPCQ. Hospital Regional de São José, Santa Catarina, Brazil, 2017. (n = 280)

The Kaiser-Guttman criterion of latent dimensions minimized the correlation between factors. The highest percentage of the variance shared by the original questions formed the first one, and those five factors would explain 67.4% of this variance. The Scree Plot demonstrated the instrument unidimensionality, as expressed in Figure 1, as well as the confirmation of the six factors defined by the original questionnaire.

**Figure 2:**
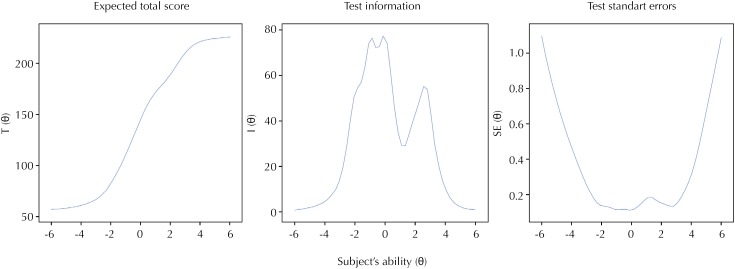
Distribution curves for the expected total score, test standard errors, and test information based on the item response theory of the Brazilian version of the QPCQ. Hospital Regional de São José, Santa Catarina, Brazil, 2017. (n = 280)

Figure 2(A) demonstrated the performance of the instrument by analyzing the angulation of the central curve, reflecting the probability of changing the response according to the individual's ability (θ). This acute angulation confirmed the strength of the QPCP to differentiate subjects according to their skill to give more precise answers. Figure 2(B) also demonstrated a symmetric curve's peak among individuals with intermediary abilities (θ). The data were not widely dispersed and no respondents had an estimated quality of prenatal level below or above the extremes on the scale. The mean quality of prenatal referred was close to the mean of the subject's skills, located between −4 and 4. Figure 2(C) expresses the test standard inconsistency according to the same ability (θ), by the symmetry and the curve's valley. The standard errors of latent skill estimates varied between 0.17 and 0.48, indicating a good precision of the estimates. More than half of respondents had quality of prenatal care above the value of the mean of the scale (zero).

**Figure 3:**
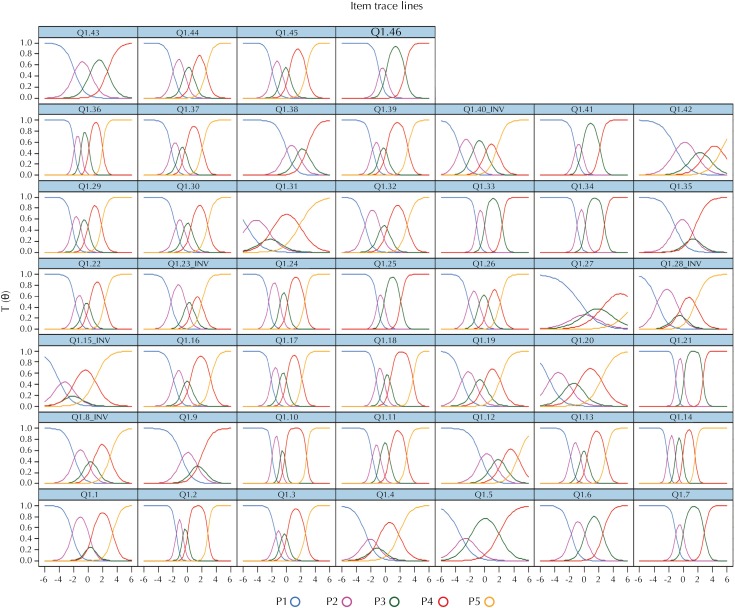
Distribution curves for the possible answers of all items of the Brazilian version of the QPCQ, based on the item response theory. Hospital Regional de São José, Santa Catarina, Brazil, 2017. (n = 280)

Figure 3 expresses, individually, the item trace lines of all items. The trace lines can be expressed numerically by the discrimination and difficulty parameters, and they are demonstrated in Table 2. The IRT demonstrated that the item 14 had the strongest discrimination (4.786), while the item 27 gave the minor contribution (0.737). Also, item 15 presented the high difficulty level (2.153), while the lower level was identified by item 12 (-2.666). These results refuted the null hypothesis that some item could be excluded from questionnaire (p < 0.001).

Considering the six factors proposed by the original questionnaire, IRT showed discrimination strength of the items grouped in factors Information Sharing, Anticipatory Guidance, Sufficient Time were similar (2.233 to 2.591). The dimensions Approachability and Availability demonstrated lower discrimination (1.540 and 1.605, respectively), differently from Support and Respect, which showed the highest mean level among factors (3.002).

## DISCUSSION

All 280 patients were Brazilian and, consequently, Portuguese was their native language. This strengthened the information found in the study, which is especially important given the cultural miscegenation in a continental country such as Brazil. Another strong point is that the study took place in a reference maternity hospital of the public health system, which receives pregnant women from a region that includes 22 counties, with more than one million inhabitants.

The 15 women considered losses or refusals had similar characteristics to those maintained in the study, however, this information was not included in the analysis and could interfere, even briefly, with the results. The sociodemographic and obstetric characteristics of the participants were very similar to those found by the Canadian^20^, Australian^21^ and French^22^ studies. Pregnancy and birth outcomes did not influence the patients’ response to the performance of the instrument.

To demonstrate the internal consistency, we evaluated the instrument overall, with Cronbach's alpha 0.975 and by factors, varying from 0.778 to 0.938. In addition, the fact that all 46 items showed Cronbach's alpha higher than 0.3 did not interfere with the final result, considered that any item were discarded. The overall Cronbach's alpha was similar to those found in the Canadian^20^ (0.910), Australian^21^ (0.970) and French^22^ (0.970) analyses. The first five factors showed higher indexes than those found in the Canadian analysis, while the sixth factor was slightly lower than the original result (0.930). The item “I was linked to programs in the community that served me” (number 27), presented the least impact in the overall evaluation, resulting in a lower reliability compared with the questionnaire. The reasons for this interpretation were unclear, since the information should be used as a quality marker for monitoring the pregnant woman.

Intraclass correlation coefficient (R = 0.995) showed excellent reproducibility (R ≥ 0.75), confirming its reliability by the consistency of measurements made by the same instrument at different times. The result was even higher than that that the questionnaire authors found, in the Canadian^20^ population [R = 0.810 (95%CI 0.760-0.850)]. Parallel reliability analysis was not performed due to the absence of another instrument capable of measuring the quality of prenatal care, validated for the Portuguese language in the Brazilian culture. Even so, this was precisely the reason for this study. Another particularity is that the confirmation of the six factors corroborated the results found in the studies performed in Canada^20^, Australia^21^ and France^22^.

The null hypothesis was refuted, because the results showed all items were suitable, due to the discriminatory capacity and degree of difficulty of the items. Therefore, the instrument presented good overall performance. Besides, it reinforces the minimum necessary load to all items remain in the instrument.

The characteristic curve of the items can describe the main theoretical assumptions of the IRT model. In this analysis, the psychological phenomenon of the latent trace, which represents the quality of prenatal care, measured by the instrument (θ) informed the ability to discriminate the item, its difficulty and the random chance of answering. The best results were found in the items (14, 21, 10, 02 and 36) with the curious fact that they all have in common the patient's perception, regarding the psychological relationship with the health care provider. Observing these items, we can infer that respondents submitted to a low-quality prenatal care were more likely to reach category number one, those who received high quality's prenatal care possibly would mark item 5. And, consequently, the remaining would represent numbers two to four in the Likert scale. On the other hand, the five worst results were identified among items related to technical issues (27, 31, 5, 42 and 20). The curves show these items demonstrate low capacity to discriminate the ability of patients who answered the questionnaire.

The expected total score curve presented high angulation. It means that even with few differences between the prenatal quality, a big variance between the probability of the answers occurred. The standard error curve demonstrated small discrepancy of the answers among those who present intermediate abilities. The test information curve indicates that the Brazilian-version of QPCQ is a value instrument, confirming the performance probability of a subject. So, few differences in the subjects’ ability (θ) determine large variations in the overall result, between intermediate abilities. On the other hand, individuals with extreme abilities present expected responses at the extremities of the Likert scale.

The discrimination parameter represented how much an item discriminated between the respondents of different skill levels, determining the quality of the item. It means that items 27 and 31 demonstrated the worst strength to discriminate between the superior and inferior quality of prenatal care.

The difficulty parameter referred to the probability of a common individual with a certain level of the latent trait selecting a category of response or a higher ordered category. This parameter represented the point on the ability scale in which there was a 50% chance that a given response category or a higher ordered category would be selected, representing the thresholds between response categories. As a wide variation between positive and negative results occurred, we verified the presence of 58% of more satisfactory and 42% of less satisfactory items regarding the degree of difficulty of the item.

This analysis allowed concluding that the Brazilian version of the QPCQ is a high quality, reliable, valid questionnaire to determine the quality of the prenatal care among Brazilian women. The questionnaire is suitable for the represented cultural context. We can also suggest the instrument presents capacity to differentiate small subtleties, in patients’ perception of the received care during pregnancy. It remains to be seen whether the Brazilian version of the QPCQ presents the same construct characteristics when applied to different population subgroups.

In Brazil, the existing instruments to verify the quality of prenatal care use technical criteria. The Brazilian version of the QPCQ allows evaluating questions on respect, attention, trust and relationship between assisting professionals and patients. This process of translation and validation allowed identifying the soundness of this instrument, as evidenced in other cultural contexts with the original questionnaire. We can infer that one can repeat and apply this process in other languages, contributing to the evaluation of a quality prenatal all around the distinct cultures.
